# Supplementary material for: The Initial Cardiac Tissue Response to Cryopreserved Allogeneic Adipose Tissue-Derived Mesenchymal Stromal Cells in Rats with Chronic Ischemic Cardiomyopathy
Source: Int J Mol Sci. 2021 Oct 29;22(21):11758. doi: 10.3390/ijms222111758 (PMC8583910; doi:10.3390/ijms222111758)
Supplement: Supplementary file 1 [file ijms-22-11758-s001.zip › ijms-1392769-supplementary.pdf]

Supplementary Material

Figure S1. Verification of injection method

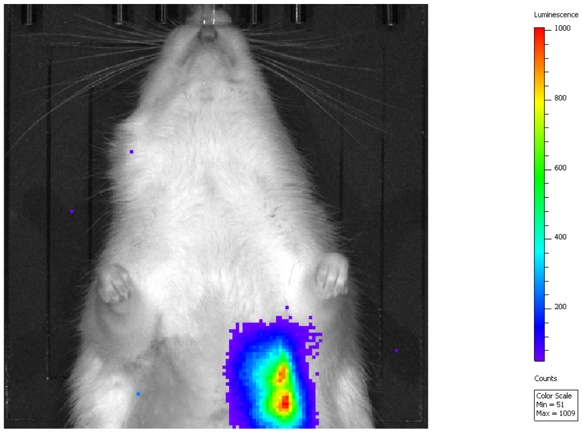

Bioluminescent image of thoracic located ASCs 24 hours after echo-guided trans-thoracic intra-myocardial injection

Figure S2. Transcriptional comparison of saline day 7 and ICM.

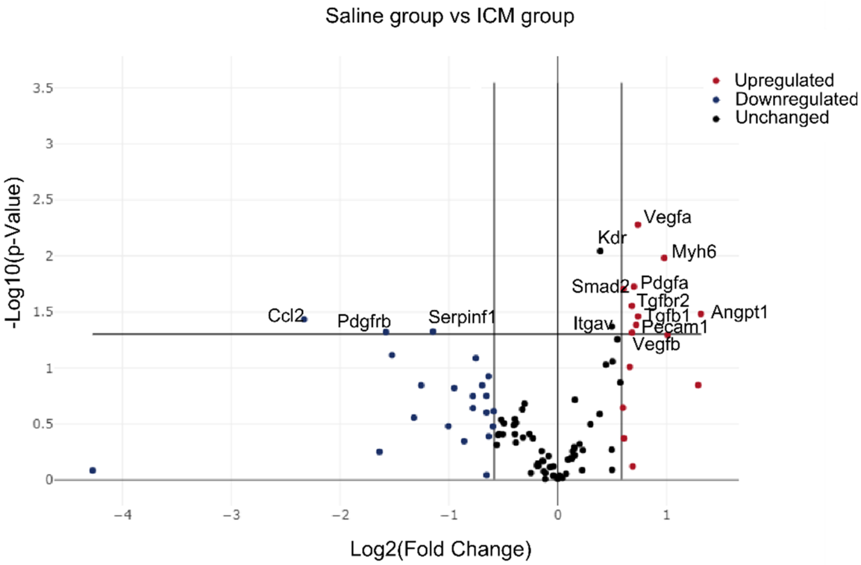

Volcano plot for comparison between saline group day 7 and ICM (n = 3 and 5, respectively).

Table S1. List of genes and controls in the RT² Profiler PCR Arrays.

Gene symbol	Description
Acta2	Smooth muscle alpha-actin
Actb	Actin, beta
Ang	Angiogenin, ribonuclease A family, member 1
Angpt1	Angiopoietin 1
Angpt2	Angiopoietin 2
Ccl2	Chemokine (C-C motif) ligand 2

Ccl3 Chemokine (C-C motif) ligand 3
Ccl5 Chemokine (C-C motif) ligand 5
Ccl7 Chemokine (C-C motif) ligand 7
Ccl9 Chemokine (C-C motif) ligand 9
Ccr1 Chemokine (C-C motif) receptor 1
Ccr2 Chemokine (C-C motif) receptor 2
Cd40lg CD40 ligand
Cebpb CCAAT enhancer binding protein beta
Mmp1 Matrix metallopeptidase 1a (interstitial collagenase)
Col1a2 Collagen, type I, alpha 2
Col3a1 Collagen, type III, alpha 1
Col4a3 Collagen, type IV, alpha 3
Csf1 Colony stimulating factor 1 (macrophage)
Csf2 Colony stimulating factor 2 (granulocyte-macrophage)
Ctgf Connective tissue growth factor
Cxcl12 Chemokine (C-X-C motif) ligand 12 (stromal cell-derived factor 1)
Cxcl13 Chemokine (C-X-C motif) ligand 13
Cxcl16 Chemokine (C-X-C motif) ligand 16
Cxcl3 Chemokine (C-X-C motif) ligand 3
Cxcl9 Chemokine (C-X-C motif) ligand 9
Cxcr3 Chemokine (C-X-C motif) receptor 3
Cxcr4 Chemokine (C-X-C motif) receptor 4
Ackr3 Chemokine (C-X-C motif) receptor 7
Fgf1 Fibroblast growth factor 1
Fgf2 Fibroblast growth factor 2
Gapdh Glyceraldehyde-3-phosphate dehydrogenase
Gata4 GATA binding protein 4
Hgf Hepatocyte growth factor
Ifng Interferon gamma
Il10 Interleukin 10
Il11 Interleukin 11
Il1b Interleukin 1 beta

Il4 Interleukin 4
Il6 Interleukin 6
Il6r Interleukin 6 receptor
Itgav Integrin, alpha V
Itgb3 Integrin, beta 3
Kdr Kinase insert domain receptor
Ltbp1 Latent transforming growth factor beta binding protein 1
Mmp14 Matrix metallopeptidase 14 (membrane-inserted)
Mmp19 Matrix metallopeptidase 19
Mmp2 Matrix metallopeptidase 2
Mmp3 Matrix metallopeptidase 3
Mmp9 Matrix metallopeptidase 9
Myh6 Myosin, heavy chain 6, cardiac muscle, alpha
Myh7 Myosin, heavy chain 7, cardiac muscle, beta
Ngf Nerve growth factor (beta polypeptide)
Nkx2-5 NK2 transcription factor related, locus 5 (Drosophila)

Nos3 Nitric oxide synthase 3, endothelial cell
Nppa Natriuretic peptide precursor A
Nppb Natriuretic peptide precursor B
Nrg1 Neuregulin 1
Pdgfa Platelet-derived growth factor alpha polypeptide
Pdgfb Platelet-derived growth factor beta polypeptide (simian sarcoma viral (v-sis) oncogene homolog)
Pdgfra Platelet derived growth factor receptor, alpha polypeptide
Pdgfrb Platelet derived growth factor receptor, beta polypeptide
Pecam1 Platelet/endothelial cell adhesion molecule 1
Plat Plasminogen activator, tissue
Polr2a Polymerase (RNA) II (DNA directed) polypeptide A
Ptgs2 Prostaglandin-endoperoxide synthase 2
Rpl4 Ribosomal protein L4
Serpinf1 Serpin peptidase inhibitor, clade F (alpha-2 antiplasmin, pigment epithelium derived factor), member 1
Smad2 SMAD family member 2
Stat1 Signal transducer and activator of transcription 1
Tlr4 Toll-like receptor 4
Tek TEK tyrosine kinase, endothelial
Tgfa Transforming growth factor alpha
Tgfb1 Transforming growth factor, beta 1
Tgfbr2 Transforming growth factor, beta receptor II
Thbs2 Thrombospondin 2
Tie1 Tyrosine kinase with immunoglobulin-like and EGF-like domains 1
Timp1 TIMP metallopeptidase inhibitor 1
Timp2 TIMP metallopeptidase inhibitor 2
Timp3 TIMP metallopeptidase inhibitor 3
Tlr2 Toll-like receptor 2
Tlr3 Toll-like receptor 3
Tnf Tumor necrosis factor (TNF superfamily, member 2)
Tnfaip6 Tumor necrosis factor alpha induced protein 6
Tnfsf13 Tumor necrosis factor (ligand) superfamily, member 13
Tymp Thymidine phosphorylase
Vegfa Vascular endothelial growth factor A
Vegfb Vascular endothelial growth factor B
Vegfc Vascular endothelial growth factor C
Cx3cr1 Chemokine (C-X3-C motif) receptor 1
Tbp TATA box binding protein
NTC No template control
NRT No reverse transcriptase control
RTC Reverse-transcription control
PPC Positive PCR control
GDC Genomic DNA control
